# 

**Published:** 1887-01

**Authors:** 


					﻿SUBSCRIPTION PRICE, $3.00 PER YEAR, IN ADVANCE.
Vhole Nutnbej>?75
JANUARY, 1881
Number 1.
TZHZZE
CHICAGO MEDICAL JOURNAL AND EXAMINER
Editor: S. J. JONES, M.D., LL.D.
PUBLISHED MONTHLY BY
THE CLARK LOKGLEY COMPANY,
Under direction of
THE CHICAGO MEDICAL PRESS ASSOCIATION,
308-316 Dearborn Street.
				

